# Impact of irrelevant speech and non-speech sounds on serial recall of verbal and spatial items in children and adults

**DOI:** 10.1038/s41598-025-85855-w

**Published:** 2025-01-14

**Authors:** Larissa Leist, Thomas Lachmann, Maria Klatte

**Affiliations:** 1 Center for Cognitive Science, Cognitive and Developmental Psychology Unit, University of Kaiserslautern-Landau (RPTU), 67663 Kaiserslautern, Germany; 2 Centro de Investigación Nebrija en Cognición (CINC), Universidad Nebrija, 28007 Madrid, Spain; 3 Brain and Cognition Research Unit, Faculty of Psychology and Educational Sciences, University of Leuven (KU), 3000 Leuven, Belgium

**Keywords:** Human behaviour, Psychology

## Abstract

Short-term memory for sequences of verbal items such as written words is reliably impaired by task-irrelevant background sounds, a phenomenon known as the “Irrelevant Sound Effect” (ISE). Different theoretical accounts have been proposed to explain the mechanisms underlying the ISE. Some of these assume specific interference between obligatory sound processing and phonological or serial order representations generated during task performance, whereas other posit that background sounds involuntarily divert attention away from the focal task. To explore the roles of phonological processing, serial order retention, and attention control, we analyzed the effects of environmental non-speech sounds and unfamiliar speech on serial recall of verbal items (pictures representing German nouns) and spatial items (dot locations) in children (n = 137) and adults (n = 98). In the verbal task, both age groups were equally affected by background sounds, with speech impairing recall more than environmental sounds. In the spatial task, no ISE was found in adults and fourth graders, but third graders exhibited significant performance impairment from both sounds. There was no habituation to the sound effects across the experimental trials. The findings indicate that both specific interference and attention capture may contribute to the ISE, with the impact of attention capture potentially decreasing with age.

## Introduction

In our daily lives, we are frequently exposed to environmental noise, such as traffic noise, music, and other peoples’ conversations. Mental work in noisy environments is becoming increasingly prevalent in our digitalized society as people are taking greater advantage of the opportunity—or the obligation—to work virtually anywhere, e.g., at home, in cafés, on trains and airplanes. The issue of environmental noise is also increasingly important for children. For them, learning in classrooms is often accompanied by task-irrelevant sounds from classmates’ activities or noise pollution from outside, which has been shown to have a negative impact on mental performance and well-being^1–4^. To avoid or reduce such impairments, we need to know more about the mechanisms underlying noise effects in different age groups. Therefore, in the present study we investigated the effects of different types of environmental noise on the short-term memory performance in children and adults.

Research on noise-induced impairments of short-term memory goes back more than 50 years, and has focused on the so-called “Irrelevant Sound Effect” (ISE). The ISE refers to the impairment of short-term memory for verbal items (e.g., digits, words, letters) presented visually due to task-irrelevant background sounds that participants are instructed to ignore. The usual paradigm for studies on the ISE uses a standard short-term memory task which requires immediate serial recall of lists of 5 to 9 items presented in succession for about one second each. The ISE is a robust and reliable phenomenon which has been replicated numerous times using a wide range of materials, designs, and procedures^5–10^. There is widespread agreement that both speech and non-speech sounds have a detrimental impact on verbal serial recall in adults.

Several theoretical frameworks have been proposed to explain the mechanisms underlying the ISE. Some of these emphasize automatic, content- or process- based interference between sound characteristics and specific task demands, whereas others attribute the ISE to more general diversions of attention away from the focal task (for a comprehensive review, see Leist et al.^11^).

Early studies performed by Baddeley and his colleagues^5,7,12,13^ focused on speech as irrelevant sound and attributed its detrimental effect to interference between phonological representations in the phonological loop component of working memory^14^. The phonological loop is a storage system for verbal information. It consists of two parts: a phonological store, which holds phonological representations in a passive form, and an articulatory rehearsal process, which prevents trace decay in the store through subvocal repetitions of the to-be-remembered (TBR) list items. According to the phonological loop account, the ISE results from specific (content-based) interference between the phonological codes of the visually presented items and the irrelevant speech that gains automatic access to the phonological store. Consequently, sounds lacking speech-specific properties such as tones or instrumental music should not impair verbal short-term memory, and tasks requiring short-term memory for nonverbal items should be unimpaired by irrelevant speech.

However, a number of later findings did not support these predictions. First, it was shown that the ISE is also evoked by reversed speech^15–18^, tones^10,19^, and music^20–23^, even though the disruption is most pronounced with speech^24^. Second, the disruptive effect of background speech is independent from its phonological similarity to the list items^25^, which contradicts the assumption that similarity between the irrelevant stimuli and the TBR items determines the magnitude of the disruption. Third, the phonological loop account was unable to explain findings demonstrating a dominant role of seriation in ISE evocation. Concerning task characteristics, verbal memory tasks without a serial component were shown to be unaffected or only mildly affected by irrelevant sounds^26^, whereas a nonverbal task requiring the recall of spatial locations in serial order was significantly affected, see Jones et al.^27^ (Exp. 4). Concerning sound characteristics, it was shown that sounds representing coherent sequences of distinct, different auditory tokens (“changing-state” sounds, e.g., sequences of different syllables produced by a single speaker) produce stronger impairments when compared to continuous, unstructured sounds like broadband noise or sounds consisting of single, repeated tokens (“steady-state” sounds, e.g., repetitions of single syllables or tones). Aiming to integrate these findings, Jones et al.^28^ provided another explanation of the ISE. According to this “changing-state account”, sounds with a changing-state characteristic are automatically structured and organized into streams of ordered objects via pre-attentive auditory streaming processes. Sequential information generated during this process interferes with the serial rehearsal of the list items that participants rely on during serial recall tasks. Thus, in contrast to “interference-by-content” proposed by the phonological loop account, the changing-state account assumes interference-by-process (seriation) as the mechanism underlying the ISE.

The changing-state account has been integrated into a broader theoretical framework of serial recall and respective impairments, which assumes that serial order retention is not accomplished through specialized memory structures or processes, but through general-purpose motor and perceptual functions (perceptual-gestural view of short-term memory, see^29,30^). According to this model, ordered recall of unrelated items is achieved through a deliberate rehearsal process that involves mapping the perceived item sequence to a motor output plan, a record of which is then cyclically repeated until recall is completed. This holds for items of any origin or modality, as they all share a common representational space. Therefore, this model predicts equivalent effects of speech and non-speech changing-state sounds, and equivalent effects of changing-state sounds on serial recall of verbal and nonverbal (e.g., spatial) items.

Neither the phonological loop nor the changing-state account incorporates a specified role of attention in ISE evocation. In contrast, Cowan’s Embedded Processes Model^31,32^ emphasizes the role of attention in memory performance and its impairments. Cowan^31,32^ posits that memory representations vary with respect to activation. Working memory is considered as a subset of long-term memory, comprising those representations that are currently in a heightened state of activation. Representations with the strongest activation enter the so-called “focus of attention”, which is limited in capacity to about four items. Maintaining a longer sequence of items is achieved by recirculating them through the focus of attention. This mechanism is termed “attentional refreshing”, and is available for verbal as well as nonverbal items. According to the attentional account, refreshing is impaired through involuntary intrusions of task-irrelevant sounds into the focus of attention, leading to poorer recall.

Aiming to explore the role of attention in the ISE, several studies compared the effects of irrelevant sounds between children and adults. Given their immature attention control, children are likely to be less able than adults to focus attention on the task in the presence of distracting stimuli. Thus, if the ISE were to result from attention capture, children should be more affected than adults. However, the findings on developmental change in ISE magnitude are inconsistent. While some studies^11,33–35^ identified a larger ISE in children when compared to adults, others found no differences between groups^35–40^.

Another approach to examine the impact of attention capture is to investigate the magnitude of the ISE over the time-course of task completion. According to the attentional account, the ISE should diminish with time, as participants habituate to the irrelevant sound with repeated exposure. Research in this area also revealed mixed results. Some studies provided evidence for habituation (and thereby attention capture)^41,42^, while others did not^43,44^. Researchers have attributed the inconsistency in habituation effects to variations in methodologies.

Especially, it has been suggested that habituation effects are more pronounced when the same sound sequence is presented in each trial of the respective condition (specific habituation) when compared to paradigms where a sound condition is represented by different sound sequences, e.g., different sequences of syllables representing a changing-state condition (unspecific habituation)^45^. According to this view, unspecific habituation proceeds more slowly and is thus not always observable over the time course of a typical ISE experiment.

In the present study, we aim to get further insight into the roles of phonological interference, seriation, and attention control in the ISE. For this aim, we investigate the effects of environmental sounds and narrative speech on serial recall of verbal and nonverbal (spatial) items in children and adults. As previously stated, in contrast to the assumption of phonology-based interference, Jones et al.^27^ reported similar effects of changing-state speech on verbal and spatial serial recall, and attributed the disruption to interference between pre-attentive seriation processes involved in obligatory sound processing, and deliberate serial rehearsal of the list items. However, the finding of an ISE in spatial serial recall could not be reproduced in several subsequent studies^11,46–50^. One of these studies^11^ compared performance of children and adults, and found a significant impairment due to changing-state speech (spoken syllables) in the verbal task, which was more pronounced in the children than in the adults. For the spatial task, no sign of a sound-induced disruption was found in either group. The latter finding is in contrast to both the changing-state and the attention-capture account of the ISE, as both assume domain-general maintenance mechanisms as the locus of the ISE.

In this study, we used narrative unfamiliar speech and a mixture of environmental non-speech sounds such as dogs barking, telephone ringing, and traffic sounds as task-irrelevant background sounds. For both sound conditions, the same sequence was used in each of the respective trials, thereby increasing the probability to detect potential habituation effects^45^. Using nonspeech environmental sounds, we deviate from prior studies, which analyzed the role of attention in the ISE by using auditory deviants (sounds that violate expectations, i.e., a rarely presented “deviant” sound within a sequence of repeated “standard” sounds, with deviation defined by acoustic properties like timbre^51^, or a change in semantic category^52^), taboo words^53,54^, or self-relevant words (e.g., the name of the participants^55^) as irrelevant sounds, and/or by varying the predictability of the respective sequences^56,57^. These studies provide evidence that irrelevant sounds may impair visual-verbal serial recall performance in adults through attention capture.

We decided for environmental sounds for several reasons. First, performing mental task in the presence of irrelevant environmental sounds is a daily challenge in working environments such as classrooms and open-plan offices. Second, sequences of different environmental sounds should evoke only a minor or no changing-state effect due to their lack of stream coherence. Especially, following the changing-state account, in order to evoke interference with seriation, an irrelevant sound sequence should represent a coherent stream consisting of different auditory tokens emanating from a single source, e.g., a sequence of different syllables uttered by a single speaker, or a melody played by a single instrument. Detrimental effects evoked by a sequence of unrelated environmental sounds may thus be considered as indicators of attention capture. Third, evidence from field studies^2,58^ and from behavioral and EEG experimental studies^36,59,60^ confirms that environmental sounds capture attention, especially in children. One of the studies^36^ explored age differences in the ISE and found that non-speech classroom noise impaired visual-verbal serial recall performance in 7-year-old first graders, whereas older children and adults were unaffected.

In light of the existing research, the following predictions arise: if attention capture plays a dominant role in the ISE, the speech and environmental sounds should evoke significant disruption in both tasks, with stronger effects in children when compared to adults. Furthermore, we should observe a decrease of disruption over the time-course of task completion due to specific habituation. Concerning the interference-based accounts, the following predictions can be derived. If the ISE results from phonological interference (Phonological Loop Account), task-irrelevant speech should impair performance in the verbal task. This effect should be equivalent in children and adults, and should remain stable over the course of the task. Environmental sounds should evoke a minor or no ISE in the verbal task as they lack phonological properties. The spatial task should not be impaired by any background sound, neither in the adults nor in the children, because spatial items are not processed in the phonological loop. In contrast, according to the changing-state account, speech should evoke impairments in both tasks, regardless of age. The disruptive effect should remain stable over the course of the tasks. The mixture of environmental sounds should evoke no or minor disruption, as it does not represent a coherent auditory stream, but a sequence of unrelated sounds from different sources.

For the verbal task, items were displayed as colorful drawings of monosyllabic German nouns. Drawings were used instead of written words in order to rule out effects of children’s reading abilities on serial recall performance. Pictorial presentation does not alter participants’ strategies when compared to printed words^61,62^, for a review see Leist et al.^11^. We recruited children aged about 9 years, as we assume that they employ verbal rehearsal strategies when memorizing verbal items^63,64^. However, it is important to recognize that rehearsal strategies can vary significantly among younger children, influencing their recall performance and susceptibility to distractions^65,66^.

For the spatial task, items were spatial locations presented as uniform black dots at varying positions on the screen. Sequence length for adults (children) was 8 (5) for the verbal task, and 7 (5) for the spatial task. Items were presented one-by-one on the computer screen with an onset-to-onset rate of 2 seconds. After a 5 seconds rehearsal interval, all items presented in the respective trial were shown in random arrangement on the screen. For serial recall, participants clicked on the items in the order of presentation. The experimental factors Task (verbal, spatial) and Sound condition (silence, environmental sounds, speech) were varied between and within participants, respectively (i.e., each participant performed either the spatial or the verbal task in each of the three sound conditions). A recording of a female speaker reading a newspaper article in a language unfamiliar to all participants was used as irrelevant speech.

## Results

**Fig. 1:**
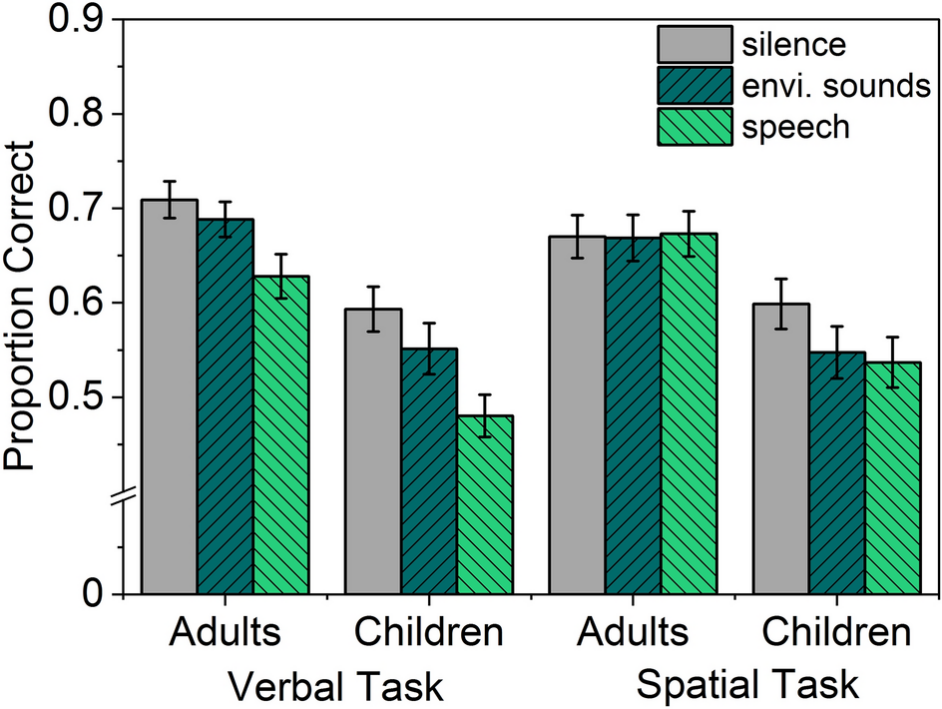
Mean proportion correct scores of both the verbal and the spatial task with respect to age group and sound condition. Error bars denote standard errors of the mean.

An item was scored as correct when it was recalled in the correct serial position^51,67^. All analyses are based on proportion correct scores. Figure 1 illustrates the proportion of correct answers based on age group and sound condition.

The data were analyzed using a 3 × 2 × 2 mixed-design Analysis of Variance (ANOVA) with Sound condition (silence, environmental sounds, speech) as within-subject factor and Age group (children, adults) and Task (verbal, spatial) as between subject factors. The analysis revealed significant main effects of Sound condition, *F* (2,462) = 24.770, *p* < .001, ${\eta }_{p}^{2}$= .097, and Age group, *F*(1,231) = 27.871, *p* < .001, ${\eta }_{p}^{2}$= .108. The main effect of Task was not significant, *F* (1*,*231) < 1. Importantly, there were significant two-way interactions between Sound condition × Task, *F*(2,462) = 8.762, *p* < .001, ${\eta }_{p}^{2}$= .037, and between Sound Condition × Age group, *F*(2,462) = 3.889, *p* = .021, ${\eta }_{p}^{2}$= .017, demonstrating a stronger effect of sound in the verbal when compared to the spatial task, and a stronger effect of sound in children when compared to adults, respectively.

Performance in the silent control condition did not differ between tasks, for either the adults, (*t*(96)= 1*.*308, *p* = *.*194, *d* = *.*265), and the children, (*t*(135)= *.*160, *p* = *.*873, *d* = *.*027). Hence, within age groups, a potential difference in the ISE between tasks cannot be attributed to differences in task difficulty. Across age groups, *t*-test revealed significantly better performance of the adults in the verbal task (*t*(119)= 3*.*565, *p* < *.*001, *d* = *.*656) and a tendency for better performance in the spatial task (*t*(112)= 1*.*928, *p* = *.*056, *d* = *.*367). The interaction between Task and Age group and the three-way interaction were not significant, *F*(1*,*231) < 1 and *F*(2*,*462) < 1, respectively. To evaluate the significant two-way interactions of Sound Condition with Age group and Task, separate analyses were performed for the verbal and spatial tasks.

### Verbal task

Data were analyzed using a 3×2 mixed-design ANOVA with Sound condition (silence, environmental sounds, speech) as within-subject factor and Age group (children, adults) as between-subject factor. Mauchly’s test indicated that the assumption of sphericity had been violated, *χ*^2^(2) = 7*.*11, *p* = *.*029, therefore degrees of freedom were corrected using Huynh-Feldt estimates of sphericity (*ε* = *.*97)^68^. There were significant main effects of Sound condition, *F*(1*.*94*,*230)= 29*.*274, *p* < *.*001, 

${\eta }_{p}^{2}$ = .197, and Age group, *F*(1,119) = 19.608, *p* < 0.001, ${\eta }_{p}^{2}$= .141. The interaction was not significant, *F*(1*.*94*,*230) < 1, indicating comparable sound effects in children and adults. Bonferroni-corrected post hoc tests revealed that performance in the speech condition was lower than the silent and environmental sounds conditions (each *p* < .001). Performance in the environmental sounds condition was lower than in the silent control condition (*p* = .019 ).

### Spatial task

Proportion correct scores were analysed using a 3 × 2 mixed-design ANOVA with Sound condition (silence, environmental sounds, speech) as within-subject factor and Age group (children, adults) as between-subject factor. There were significant main effects of Sound condition, *F*(2,224) = 3.394, *p* = .035, ${\eta }_{p}^{2}$= .029, and Age group, *F*(1,112) = 9*.*787, *p* = .002, ${\eta }_{p}^{2}$= .080, and a significant interaction, *F*(2,224) = 3.394, *p* = .025, ${\eta }_{p}^{2}$= .032, indicating that the sound effect differed between age groups.

Separate analyses were performed for each age group in order to analyze the significant interaction. A one-way repeated measures ANOVA of the factor Sound condition (silence, environmental sounds, speech) did not reach significance for the adults, *F*(2*,*92) < 1, but revealed a significant main effect for the children, *F*(2,132) = 6.205, *p* = .003, ${\eta }_{p}^{2}$= .086.

Post hoc Bonferroni-corrected tests of the children’s spatial task performance revealed lower performance in the speech condition (*p* = .006) and in the environmental sounds condition (*p* = *.*030) when compared to silence. The sound conditions, environmental sounds and speech, did not differ (*p* = 1*.*00).

**Fig. 2:**
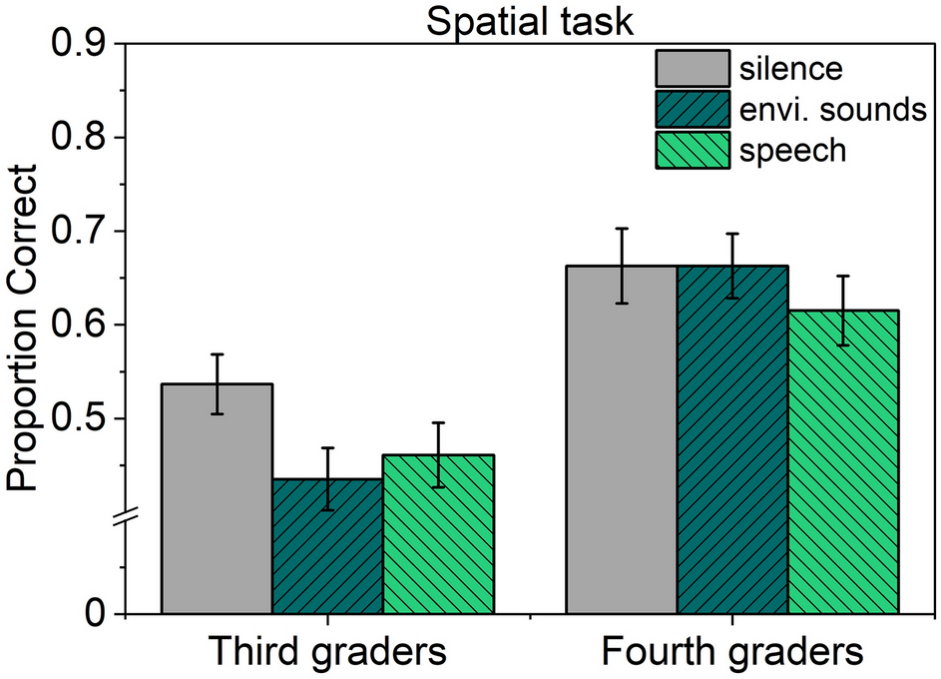
Mean proportion correct scores of the spatial task with respect to class level and sound condition. Error bars denote standard errors of the mean.

The significant sound effect in children is in contrast to Leist et al.^11^, who reported no disruptive effect of sounds on children’s performance in the spatial task. However, in that study, only third graders were included, whereas in the current study, the spatial task was performed by both third and fourth graders. As attention control develops rapidly during the early years of schooling^69–73^, we conducted an additional analysis to explore the potential influence of class level on the sound effect. This analysis was performed on 34 third graders (20 female, 14 male, 7;1–9;6 years, *M* = 8;4, *SD* = .4 ) and 33 fourth graders (20 female, 13 male, 8;1–11;3 years, *M* = 9;6, *SD* = .6 ). Figure 2 illustrates the proportion of correct answers based on class level and sound condition. A 3 × 2 mixed-design ANOVA with the within-subject factor Sound condition (silence, environmental sounds, speech) and the between-subjects factor Class level (third grade, fourth grade) revealed significant main effects of Sound condition, *F*(2,130) = 6.400, *p* = .002, ${\eta }_{p}^{2}$= .090, and Class level, *F*(1,65) = 14.141, *p* < .001, ${\eta }_{p}^{2}$= .179, and a significant interaction, *F*(2,130) = 4.053, *p* = .020, ${\eta }_{p}^{2}$= .059. Separate repeated-measures ANOVAs revealed a significant effect of Sound condition for the third-graders, *F*(2,66) = 8.957, *p* < .001, ${\eta }_{p}^{2}$= .213, but not for the fourth-graders, *F*(2,64) = 2.061, *p* = .136, ${\eta }_{p}^{2}$= .060.

### Analyses concerning habituation

**Fig. 3:**
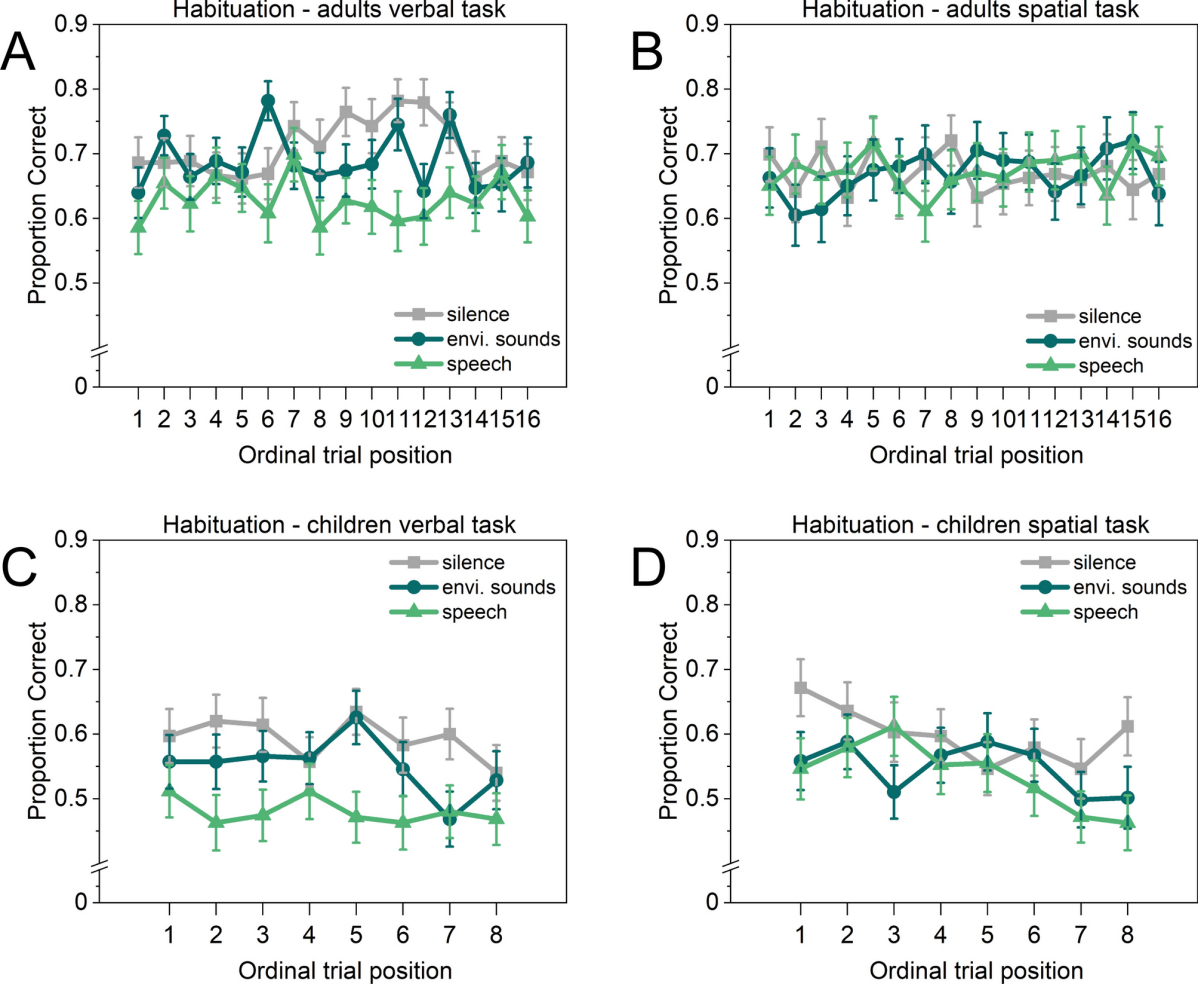
Mean proportion correct scores as a function of sound condition and ordinal trial position of sixteen (adults) and eight (children) trials for both tasks (verbal, spatial). Error bars denote standard errors of the mean.

To investigate the impact of habituation on the ISE, an additional analysis was conducted to determine whether the sound induced impairments revealed in the preceding analyses diminish over time. If the sound effects are caused by attention capture, one might expect that the first trials with sound would be more disrupted than the last ones, i.e., over time, participants become habituated to the repeated sound sequence (specific habituation, see Röer et al.^45^). Proportion correct scores were calculated for each age group and task for the ordinal trial position, with sixteen trials (adults) and eight trials (children) for each sound condition. Figure 3 depicts the resulting proportion correct scores.

Two-factor repeated measures ANOVAs were performed with Sound condition (silence, environmental sounds, speech) and Trial (1–16) for adults, and Trial (1–8) for children, separately for each task (verbal, spatial).

The analysis for the adults’ spatial task performance revealed no significant effects of Sound condition, *F*(2*,*92) < 1, *p* = .939 , Trial, *F*(15*,*690) < 1, *p* = .982, and no interaction, *F*(30*,*1380) < 1, *p* = .879 . The analysis for the adults’ verbal task performance revealed a significant main effect of Sound condition, *F*(2,100) = 16.058, *p* < .001, ${\eta }_{p}^{2}$= .243, no main effect of Trial, *F*(15*,*750) = 1*.*24, *p* = .236 , and no interaction, *F*(30*,*1500) = 1*.*410, *p* = .070. The same pattern was found for the children’s verbal task performance, a significant main effect of Sound condition, *F*(2,138) = 17.606, *p* < .001, ${\eta }_{p}^{2}$= .203, no main effect of Trial, *F*(7*,*483) < 1, *p* = .434, and no interaction, *F*(14*,*966) < 1, *p* = .633. The analysis of the children’s spatial task performance revealed significant main effects of Sound condition, *F*(2,132) = 6.205, *p* = 0.003, ${\eta }_{p}^{2}$= 0.086, and.

Trial, *F*(7,462) = 2.317, *p* = 0.025, ${\eta }_{p}^{2}$= 0.034, but no interaction, *F*(14*,*924) < 1, *p* = .456, indicating that, regardless of sound conditions, children’s overall performance in the spatial task declined over time. Thus, no evidence for habituation was found.

## Discussion

This study provides a follow-up extension of earlier research conducted by Leist et al.^11^, who showed that children’s and adults’ serial recall performance of verbal but not spatial items is impaired by task-irrelevant changing-state speech. Here, we investigated the impact of task-irrelevant speech and environmental non-speech sounds on verbal and spatial serial recall in primary school children and adults. The tasks were identical to Leist et al.^11^, requiring the serial reconstruction of pictures displaying monosyllabic German words (verbal task) and of uniform black dots displayed at various positions on the screen (spatial task).

In the verbal task, children and adults were equally affected by both task-irrelevant background sounds. Speech impaired recall performance significantly more than environmental sounds. There was no evidence of habituation across experimental trials, neither in the children nor in the adults. For the spatial task, performance of adults was unaffected by the irrelevant sounds, which is in contrast to the classical finding of Jones et al.^27^, but in line with more recent findings^11,50^. However, for children’s performance in the spatial task, background speech and environmental sounds evoked significant disruption of equal magnitude. Further analyses revealed that the significant sound effect in the child sample resulted from a reliable impairment in the third-grade children, whereas fourth graders were unaffected. The pattern of results is discussed with respect to the theoretical accounts outlined in the introduction.

In adults, the ISE was evident for visual-verbal serial recall, but was completely absent for serial recall of spatial items. Given the consistent evidence from the current and other studies with considerable statistical power^11,50^, we may conclude that serial recall of spatial items is unaffected by irrelevant sounds in adults. This poses severe difficulties for theories positing that the ISE results from impairments of domain-general maintenance mechanisms, i.e., perceptual-motor sequencing (changing-state account) and attentional refreshing (attention-capture account), as both predict sound-induced performance decrements irrespective of item modality. Importantly, according to the attention capture account, ISE should even be stronger for spatial when compared to verbal serial recall, as it has been shown that visuo-spatial working memory tasks rely much more on domain-general attentional resources^74,75^. The immunity of spatial serial recall to the ISE is in line with the phonological loop account, which considers the retention of phonological codes in a verbal short-term store as the locus of disruption.

The finding that environmental non-speech sounds also impaired verbal serial recall performance, albeit to a lesser extent than speech, mirrors the results reported in Buchner et al.^76^. These authors explain their findings in the framework of the changing-state account, arguing that speech sounds have a more prominent changing-state characteristic when compared to environmental sounds. In particular, according to the changing-state account, speech is more disruptive than non-speech sounds because it represents a coherent changing-state auditory stream, whereas the sound mix consists of isolated and unrelated auditory tokens. However, the difference in effect magnitude between speech and environmental sounds might also be explained by the phonological loop account, by assuming that complex non-speech sounds are processed in the phonological loop, as was suggested for instrumental music^7^, or that environmental sounds—like spoken words—automatically activate phonological representations which interfere with those of the list items. Recent findings suggest that spoken words and environmental sounds evoke comparable phonological activation^77^, but see Kukona et al.^78^ for contradicting results. For the attention capture account, the reduced effect of environmental sounds is difficult to explain. One would expect that a mixture of heterogeneous, partly alarming sounds has more potential to attract participants’ attention when compared to narrative, meaningless speech. In addition, the attention capture account predicts habituation with repeated exposure, which was not found for either sound.

Concerning developmental change, the absence of age-related differences in the ISE on the verbal task is in line with the interference-based accounts, as both predict comparable effects across age groups provided that the same strategies are applied. The lack of an age effect is difficult to handle for the attentional account. Due to the immature attention control in children, they should be more prone to sound-induced attention capture. However, the current results need to be considered in the context of prior studies on developmental change in the ISE on verbal serial recall, which yielded inconsistent results. Some mirrored the current finding of equivalent impairments in children and adults^35–40,79^, whereas others found stronger impairments in the children^11,33–35^, thereby confirming a role of attention in the ISE. To account for these discrepancies, following Klatte et al.^36^ and Elliott et al.^35^, we suggest that children and adults are equally prone to specific interference through task-irrelevant sounds in visual-verbal serial recall (provided that the children use the same retention strategies), but that the disruptive effect may be superimposed by sound-induced diversions of attention. The contribution of the latter depends on characteristics of the sounds and method, and on the participants’ ability to control attention, which increases with age and schooling^71–73^. Developmental studies on the ISE differ considerably in these respects, for example concerning children’s age and class levels, types of background sounds (e.g., meaningless vs. meaningful narrative speech, environmental sounds, tones), sound levels, and methods of sound presentation (blocked vs. trial-by-trial variations of sound conditions, presentation via earphones vs. loudspeakers). All of these aspects have an impact on the sounds’ potential to grab attention away from the primary task. In addition, it has to be kept in mind that, even within a restricted age range or class level, there are huge individual differences in children’s attention control^69,70^. This might explain why, in our study, we did not find an age effect on verbal serial recall, whereas a prior study using comparable methods and age groups^11^ reported greater impairment in children compared to adults.

Concerning the spatial task, the current study revealed disruptive effects of equal magnitude due to speech and environmental sounds in children, but not in adults. We attribute the effect observed in the children to attention capture for several reasons. First, additional analyses revealed that the effect was only evident in the third-grade children, but absent in the fourth graders. In view of the increase in attention control with age and schooling, we may conclude that the fourth-grade children were better able to focus attention on the task in the presence of irrelevant sounds. In line with this argument, Klatte et al.^36^ found a significant impairment of verbal serial recall due to non-speech classroom noise in 7-year-old first graders, whereas older children were unaffected. Second, the current findings mirror those of a previous study^35^, which showed that children and adults were equally impaired by irrelevant speech in a visual-verbal serial recall task, but only the children were also affected in a verbal task that has consistently proven immune to the ISE in adults. The authors infer that children and adults are equally prone to sound-induced interference in visual-verbal serial recall, but that, in children, attentional diversions are also at play, making them vulnerable to sound-induced impairments irrespective of task demands and the sounds’ changing-state.

To summarize, none of the described theoretical accounts of the ISE is able to integrate the full pattern of results in our study. The findings are best explained by assuming that both specific interference with visual-verbal serial recall and more general effects on attention contribute to the observed effects, as suggested in the “duplex mechanism account” of auditory distraction^80^. Following this account, specific interference and attention capture represent independent mechanisms of sound-induced performance decrements, which may act in isolation or in combination, depending on characteristics of the tasks, the sounds, and the individuals affected. In children, diversions of attention have a stronger impact on the overall disruption, as children are less able to control attention in the presence of task-irrelevant stimuli when compared to adults. However, there is huge interindividual variability in children’s attention abilities over the primary school years, which may contribute to the discrepancies in results from studies on developmental change in the ISE.

Several limitations of the present study must be acknowledged. First, the children performed only 8 trials per sound condition, and we thus cannot rule out that habituation effects become evident when the time-course of exposure is extended. Second, with the environmental sounds used here, only limited evidence for performance decrements through attention capture was found in adults. However, as stated in the introduction, a number of studies found clear evidence for task impairments through sound-induced diversions of attention in adults^51–55^. Obviously, the attention-grabbing potential of the sounds used in those studies (i.e., auditory deviants, taboo-words^53,54^, self-relevant words^55^) is stronger when compared to the current environmental sounds. Third, as in the majority of respective studies, our child participants aged 8 years or more. As attention control increases strongly during the primary school years and the ability to filter out irrelevant sounds becomes adult-like with about age 10, future studies on age differences in the ISE should include younger children (school starters, aged 6 to 7 years). Fourth, due to the strong correlation between chronological age and class level (*r*(67)= *.*82), we do not know whether age or schooling is responsible for the stronger sound effect on the spatial task observed in third graders compared to fourth graders.

Experimental research on the ISE in children does not only increase our knowledge on the mechanisms underlying this effect, but may also contribute to practical issues. As verbal short-term memory plays a crucial role in language and reading acquisition^81^, sound-induced disruption could result in lasting deficits. Field studies have already demonstrated that noise in classrooms affects reading acquisition and verbal precursors of reading^1,2,4,82^. Therefore, experimental studies on the ISE in children are critical for creating suitable acoustic environments for learning^36^. For example, tasks that involve verbal short-term memory, such as reading and spelling in beginning readers, vocabulary learning, or mental arithmetic should not be performed in the presence of background speech or music.

## Methods

### Participants

An a priori power analysis was conducted using G*Power^83^ (version 3.1.9.7) to estimate the sample size based on data from Leist et al.^11^ (*N* = 164). The effect size reported in that study was 0.176. With a significance criterion of *α* = .05 and a power of .80, the minimum sample size required for the mixed ANOVA with this effect size is *N* = 108. A total of 137 children, recruited from two primary schools in the Kaiserslautern region and in North Rhine-Westphalia in Germany (76 female, 61 male, aged between 7 years, 9 months and 11 years, 3 months, mean age of 9;0 years), and 98 students from the University of Kaiserslautern-Landau (45 female, 53 male, aged between 19 and 36 years, mean age of 23.1 years), participated in this study.

The verbal task was performed by 51 adults (22 female, 29 male) aged between 19–26 years (*M* = 22*.*2 years, *SD* = 1*.*82 years) and 70 children (36 female, 34 male) aged between 8;0 years to 10 years, 9 months (*M* = 8 years, 6 months, *SD* = 7 months). The spatial task was performed by 47 adults (23 female, 24 male) aged between 19–36 years (*M* = 24*.*0 years, *SD* = 3*.*48 years) and 67 children (40 female, 27 male) aged between 7 years, 9 months to 11 years, 3 months (*M* = 9 years, 1 month, *SD* = 7 months).

All participants were native German speakers and had normal or corrected-to-normal vision and normal hearing according to self-reports (adults) or parental reports (children). Adults received either course credit or payment for participation (10 €). Children received writing materials, e.g., pencils, erasers.

### Apparatus

A 15.6-inch HP laptop (ProBook 650 G1) running Python 3.7/PsychoPy 3.1.5^84^ on Windows 10 was used to control the experimental trial. Display resolution was 1920×1080, with a refresh rate of 60 Hz. The background sounds were played via open headphones (Sennheiser HD650), connected with a Focusrite Scarlett 2i2 2nd generation audio interface to the laptop.

### Irrelevant sounds

Task performance was measured during three different sound conditions: silent control, environmental non-speech sounds, and speech. The environmental sounds encompassed a sequence of diverse common sounds (e.g., dog barking, bicycle bell, church bell, approaching car, chainsaw, cackling hens, siren horn). These environmental sounds were played one after the other, without pause, for two to three seconds per sound. The speech consisted of a female Danish speaker reading an article from a newsletter identical to Klatte et al.^36^. Danish was unfamiliar to all participants. For both sound conditions, the same sequence was played in each of the respective trials.

### To-be-remembered items

For the verbal task, for children and adults, items were drawn from a set of ten colorful drawings representing the German monosyllabic words *Bett (bed), Bus (bus), Eis (ice cream), Frosch (frog), Kamm (comb), Mond (moon), Pilz (mushroom), Schal (scarf), Schiff (ship)* and *Zaun (fence)*. For the adults the set included the additional items *Brief (letter), Haus (house), Herz (heart), Hut (hat), Nuss (nut)* and *Schwein (pig)*. Four lists of five items (drawn out of 10) and eight lists of eight items (drawn out of 16) were created for the children and the adults, respectively. The lists were created quasi-randomly, with each item appearing in one of four lists (children) or eight lists (adults). To generate parallel versions of each list, random permutations of the list items were used. In addition, one practice list for each sound condition was constructed. All drawings were presented in a rectangular black frame (102×73 mm) in the center of a white screen.

For the spatial task, items were presented as black dots (10 mm diameter) on a white background in a 304×183 mm black frame. Dot positions were drawn from a total of 80 possible locations that were arranged in a 8×10 grid (not shown to the participants) in the frame. As shown by Parmentier and collegues^85,86^, the difficulty of reproducing a sequence of dot locations depends on the complexity of the spatial path formed by the successive dots. Path complexity is determined by the number of crossings, the path length, and the angular degree. In order to verify equal task difficulty across sound conditions, we first created eight lists of five (children) and sixteen lists of seven dot positions (adults). The resulting path of each list was mirrored horizontally and vertically, thereby varying dot locations while keeping path complexity constant (for further details, see Leist et al.^11^). With this strategy, three parallel versions of each list were constructed, which were assigned randomly to the three sound conditions. In addition, three practice lists were constructed.

### Serial recall tasks

**Fig. 4:**
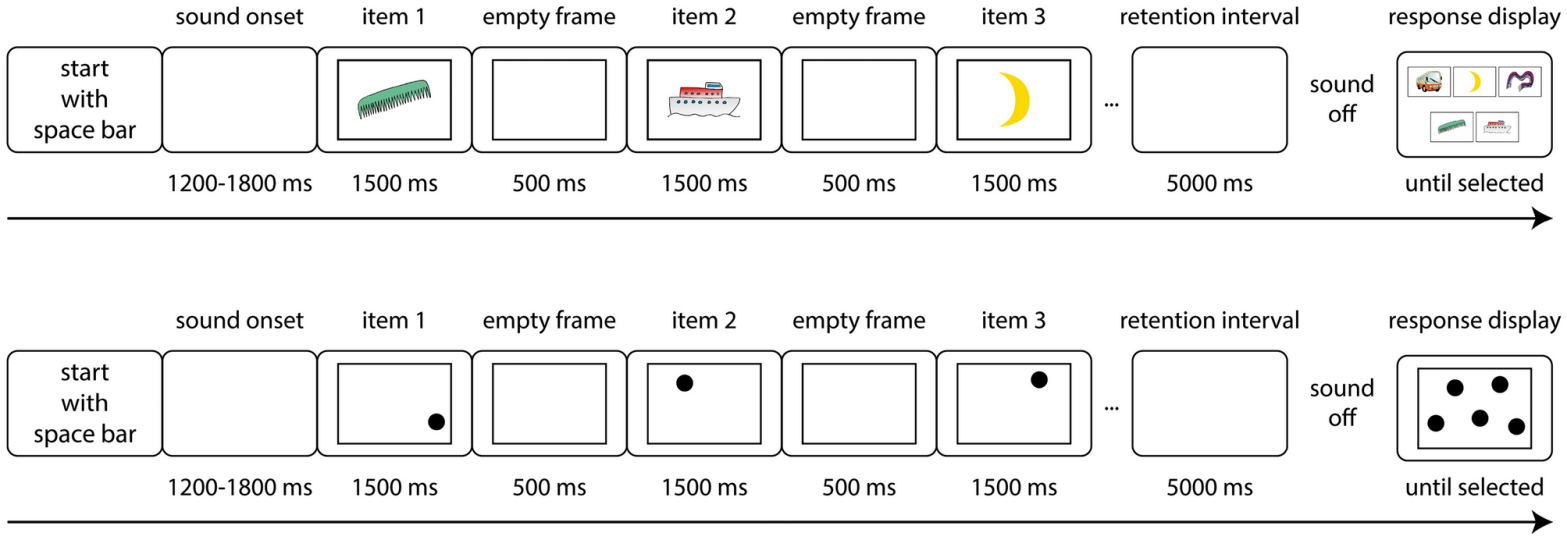
The experimental procedure for the serial recall tasks. Top: Verbal Task, bottom: Spatial Task. The children were shown five items per trial in both tasks, while the adults were shown eight (verbal task) and seven (spatial task) items per trial. The items in the verbal task were drawings, while the items in the spatial task were uniform black dots. All items shown in the relevant trial were presented again on the response display in the reconstruction phase. The pictures in the verbal task were arranged in an array of 5 (children) and 8 (adults) frames. The spatial task’s black dots were displayed in the same location as they had been shown in the trial. Note that the items are enlarged for illustrative purposes.

Both tasks are illustrated in Figure 4. Each trial consisted of a presentation phase, a retention interval, and a reconstruction phase. Items were presented one after the other with a 1500 ms presentation time and a 500 ms interstimulus interval. The final list item was followed by a 5000 ms retention interval. In the reconstruction phase, all items were displayed simultaneously on the screen. In the verbal task, drawings were randomly positioned in a predefined array of 5 (children) or 8 (adults) frames. In the spatial task, the dots were shown in the same locations as in the presentation phase. Participants were required to reproduce the serial order of drawings/dots by clicking on the items in the order in which they were presented. When an object was clicked, its shading changed to indicate that it has been selected. There was no time limit for responding and no possibility for error correction. After selecting the final item, the participants were shown a visual cue instructing them to hit the spacebar to initiate the next trial.

### Procedure

Adults were tested in two to five-person groups in a sound-attenuated booth at the University of Kaiserslautern-Landau. Children were tested individually or in groups of up to five at their school in a large classroom, where five computer workstations were set up about ten meters apart. A researcher or trained student assistant instructed each child individually. Adults received written instruction. Participants were instructed to ignore the sounds and focus on the serial recall task. For the verbal task, all pictures used were presented and named by a female speaker. To introduce the background sounds to the participants, both the environmental non-speech sounds and speech were played for 4 seconds each, followed by one practice trial per sound condition. Then, the children completed a total of 24 experimental trials, eight for each sound condition. Adults performed a total of 48 trials, 16 for each sound condition.

For both tasks, the three versions of each list were randomly assigned to the sound conditions. Sound conditions varied from trial to trial and were quasi-randomized: All sound conditions were presented in random order before being randomized again. In the trials with sound presentation, the sound started when the participant initiated the trial with the space bar. A random interval between 1200 and 1800 ms was introduced before the presentation of the first list item, to avoid correlations between sound and item onset^87^. The sound was played throughout both the presentation phase and retention interval and stopped at the beginning of the reconstruction phase. The average sound level (LAeq) of the background sounds was 59 dB, as measured by ITA Artificial Head^88^. The test session lasted about 25 minutes for children and 35 minutes for adults.

## Data Availability

The datasets generated during and analyzed during the current study as well as the materials are available in the OSF repository, https://osf.io/eayhb/?view_only=441bef7c4f5f487091b7e31f43b684dc.
